# The Story of the Insane from Year to Year

**Published:** 1900-09-29

**Authors:** 


### THE STORY OF THE INSANE FROM YEAR TO YEAR.

(Twelfth Series.)

### THE ARGYLL AND BUTE ASYLUM.

During the year 1898 the number of patients increased from 418 to 448. There were 78 first admissions, 20 re-admissions, 34 recoveries, and 23 deaths. The recoveries were 31 per cent. of the admissions, and the deaths were 4 per cent. of the average number resident. Of the deaths only 3 were from cerebral diseases, 6 from phthisis, 1 from pneumonia, and 1 from bronchitis. Moral causes account for the insanity in 2 of the year's admissions, intemperance in drink for 4, and hereditary influence for 23. The increase in the number of the patients is most unusual in this district, and curiously enough it has been almost wholly confined to Argyllshire, a fact of which Dr. Cameron says he is unable to offer any explanation. The Commissioners say that "the day rooms and dormitories were found clean and in good order," but they recommend the introduction of electric light. The weekly cost of maintenance was 8s. 8d. per head.

### THE ESSEX COUNTY ASYLUM.

On January 1st, 1898, there were 1,821 patients in residence, and at the end of the year this number had increased to 1,921. There were 615 first admissions, 174 readmissions, 294 recoveries, 70 discharged relieved, and 216 died. The recoveries stand at 37·2 per cent. of the admissions, and the deaths at 11·8 per cent. of the average number resident. Of the deaths, 72 were owing to cerebral and spinal diseases, 12 to bronchitis, 40 to pneumonia, 2 to pleurisy, 16 to phthisis, 4 to enteritis, and 1 to enteric fever. Of the year's admission, 51 owed their insanity to various moral causes such as adverse circumstances, domestic trouble, and religion; 109 to alcoholic intemperance, 150 to previous attacks, and 164 to hereditary influence. The general mortality was above the asylum average, and that from pneumonia was very high. Infectious diseases visited the asylum in the form of enteric fever, diphtheria, enteritis, and influenza. Careful examination of the drains and of the food and water supplies failed to show any cause for these diseases, and they were limited respectively to 3 and 2 cases so far as the first two diseases were concerned. Dr. Amsden must have had an anxious time, for in addition to these outbreaks one of the patients tore out his eyes and another set fire to her clothing. Temporary buildings have been put up to accommodate the additional number of patients, but these buildings will be removed when the asylum for West Ham is opened. Dr. Amsden does not take a very hopeful view of the insanity in his county. He points out that the population of Essex is rapidly increasing, and it "must be expected that we shall have a proportionate increase in the number of insane patients requiring asylum treatment." This is only too certain, and it behoves the Committee of Visitors to think what ought to be done to meet the difficulty while time is before them. The weekly charge to the unions is 9s. 4d. per head.

### THE BALLINASLOE DISTRICT ASYLUM.

Here the limits of accommodation are stated to be for 840 patients, but at the end of the year 1898, 1,086 were in residence. There were 170 first admissions, 51 readmissions, 96 recoveries, and 51 deaths. The recoveries stand at 43·4 per cent. of the admissions, and the deaths at 4·8 per cent. of the average number resident. The former of these percentages is high, and the latter very low. Of the deaths 10 were caused by cerebral and spinal diseases, 18 by consumption, and 4 by pneumonia. Of the admissions, 7 owed their insanity to moral causes, 12 to intemperance in drink, 51 to previous attacks, and 36 to hereditary influence. Extensive additions are being carried out. A detached hospital block for 260 patients was approaching completion on the date of the report, and it was proposed to remodel the laundry and build a new board-room. As usual in Irish asylums, the committee do not publish a report, and this year Dr. Fletcher's report does not convey much information. The annual cost per head was £20 13s. 3d.

### THE HANTS COUNTY ASYLUM AT FAREHAM.

On January 1st, 1898, this asylum contained 1,004 patients, and on the last day of December the number had risen to 1,043. There were 225 first admissions, 48 readmissions, 97 recoveries, and 106 deaths. The percentage of recoveries was 35·2 of the admissions, and that of the deaths was 10·3 of the average number resident, both of these being close on the asylums average for England. Thirty-eight of the deaths were caused by cerebral and spinal diseases, 11 by consumption, and 4 by pneumonia. Of the year's admissions 32 owed their insanity to various moral causes, 23 to intemperance in drink, and 44 to hereditary influence. Dr. Worthington points out that the figures relating to the causes of insanity cannot be relied on, "owing to the ignorance of the relatives who supply the information, and their wish not to divulge unpleasant secrets." Undoubtedly this is so more especially with regard to heredity and drink. Like so many others this asylum is full, indeed it contains 38 more patients than it can properly accommodate. The subject of providing additional accommodation is being considered, and it is much to be desired that no further increase be made to the old asylum. A new asylum in some other part of the county is the only correct solution of the difficulty. Dr. Worthington speaks highly of Maignen's system of softening water. If a more extended trial confirm these encomiums it will be a real boon in all those asylums which draw their water from the chalk. The Commissioners in Lunacy say that "no steps have been taken to provide means for the carrying out of the finer pathological work, which we consider to be absolutely necessary to enable the doctors to adequately discharge their medical duties." Doubtless Dr. Worthington has privately given the Committee of Visitors his opinion on this statement, but it would be interesting to have his views publicly stated, and he might append thereto the number of private practitioners in the county of Hampshire who do not engage in "the finer pathological work," and who presumably cannot "adequately discharge their medical duties." The committee "express their entire satisfaction with the able manner in which the management of the asylum is conducted by Dr. Worthington." The weekly rate of maintenance is 9s. 1d. a head.

### THE DURHAM COUNTY ASYLUM.

This asylum contained 1,433 patients on January 1st, 1898, and on the last day of the year there were 1,436 in esidence. There were 301 first admissions, 51 readmissions, 165 recoveries, 9 discharged relieved, and 173 deaths. The recoveries stand at 45·7 per cent. on the admissions, and the deaths at 11·9 per cent. on the average number resident, both of these percentages being high. Of the admissions 53 owed their insanity to various moral causes, 66 to intemperance in drink, and 75 to hereditary influence. Of the deaths, 96 were caused by cerebral and spinal diseases, 19 by pneumonia, and 19 by consumption. The asylum is practically full on the women's side, and there are not many vacancies on the male side. As the asylum is already unwieldy in size it is to be hoped that no further additions will be made to it, and the committee will recognise the necessity for building a new asylum for the county—a necessity which ought to have been seen long ago. The committee say that the management of the asylum by Dr. Smith and his subordinate officials continues to meet with their approval. The weekly cost for maintenance is 9s. 10d. per head for pauper patients, and private patients pay from 10s. 6d. to 17s.

### THE DISTRICT ASYLUM, AYR.

Here an increase from 462 to 492 has to be chronicled. There were 127 first admissions, 37 readmissions, 66 recoveries, 13 discharged relieved, and 44 deaths. The recoveries were 40 24 per cent. of the admissions, and the deaths 9·17 per cent. of the average number resident. Of the deaths, 10 were caused by cerebral or spinal diseases, 5 by bronchitis, 5 by phthisis, and 1 by pneumonia. We failed to find any table showing the causes of the insanity in the year's admissions. Dr. Skae points out that there has been an average annual increase of 5·9 per cent. in the admissions during the last ten years, and he does not think that this difference was caused by the commitment to the asylum of such cases as would formerly have been kept in their own homes; and he points out that the vast majority were acute cases. Nor can he credit the increase in the general population of the county with the increase in the insane; but he arrives at the depressing conclusion that "it would seem rather to be accounted for through the occurrence of a general increase of the usual forms of insanity." The Commissioners say that "every section of the asylum was found in excellent order," and that "there was a complete absence of excitement." The Visiting Committee do not publish their report, and we did not see any statement of the maintenance rate.

